# Incremental predictive value of deep crypts in the basal inferoseptum in the setting of hypertrophic cardiomyopathy

**DOI:** 10.1186/1532-429X-15-S1-O33

**Published:** 2013-01-30

**Authors:** Djeven P Deva, Lynne K Williams, Melanie Care, Katherine A Siminovitch, Hadas Moshonov, Bernd J Wintersperger, Harry Rakowski, Andrew M Crean

**Affiliations:** 1 Department of Medical Imaging, University of Toronto, Toronto, ON, Canada; 2 Department of Medical Imaging, St Michael's Hospital, Toronto, ON, Canada; 3 Division of Cardiology, University of Toronto, Toronto, ON, Canada; 4 Fred A. Litwin Centre in Genetic Medicine, University Health Network and Mt. Sinai Hospital, Toronto, ON, Canada; 5 Department of Clinical Radiology, University of Munich, Munich, Germany

## Background

Inferoseptal crypts have been previously reported as a marker of hypertrophic cardiomyopathy (HCM) as well as genotype positive/phenotype negative HCM. We hypothesized that the presence of such crypts would have incremental predictive value for the presence of a disease-causing mutation compared to prediction based solely on left ventricular morphologic pattern of hypertrophy.

## Methods

Cardiovascular magnetic resonance (CMR) data sets of 300 consecutive unrelated HCM patients who underwent genetic testing were reviewed independently by two experienced clinical CMR readers who were blinded to results of genetic testing. Readers assessed for presence or absence of deep basal inferoseptal crypts (DBISC) on cine steady state free precession and late gadolinium enhancement sequences and documented the imaging plane in which DBISC were convincingly visualized. Imaging and genetic testing results were correlated.

## Results

Deep basal inferoseptal crypts occurred more commonly in patients with disease-causing mutations than in patients without them (36.3% vs. 4.5%; p<0.001) and the presence of DBISC was a stronger predictor of the presence of disease-causing mutations than reverse septal curvature morphologic phenotype (p=0.025). 91.2% of patients with both reverse septal curvature and DBISC had disease-causing mutations (positive predictive value 91.2%, sensitivity 25.6%, specificity 98.3%). Mutations discovered in patients with DBISC had a higher likelihood of being disease-causing than non-disease-causing (p<0.001). DBISC were more commonly identified in patients with reverse septal curvature than in all other morphologies combined (p<0.001). In approximately half of cases, DBISC were missed on short-axis cines.

## Conclusions

Presence of deep basal inferoseptal crypts in HCM patients demonstrate incremental predictive value for genotype-positive status over that of left ventricular morphologic phenotype based prediction. Assessment for DBISC is best performed with the use of multiplanar CMR data sets. Additional imaging planes double the sensitivity for detection of deep basal inferoseptal crypts. Where genetic testing is not available, or a limited resource for economic reasons, the presence of CMR-detectable crypts may represent one factor among several that may guide decision-making for genetic testing. They may also have a role in investigating patients with modest degrees of hypertrophy (13-15mm).

## Funding

None.

**Table 1:** Incidence of hypertension, disease-causing mutations and deep basal inferoseptal crypts in the various morphologic phenotypes

	Reverse septal curvature (n=119/ 39.5%)	Apical (n=80, 26.6%)	Basal septal hypertrophy (n=73, 24.3%)	Concentric (n=17, 5.7%)	G+/P- (n=10, 3.3%)	Mass-like (n=1, 0.3%)	Non-contiguous (n=1, 0.3%)
Arterial hypertension	28.8%	35%	35.6%	58.8%	0%	0%	0%

Disease-causing mutation	66.9%	16.3%	21.9%	11.8%	100%	100%	0%

Deep basal inferoseptal crypts	29.7%	5.0%	4.1%	5.9%	90.0%	0%	0%

Maximum end-diastolic wall thickness (mm)	22.2±5.8	17.9±5.3	17.4±3.4	20.2±5.9	8.9±2.3	25.5	18.0

G+/P- denotes Genotype Positive/Phenotype Negative

**Figure 1:**
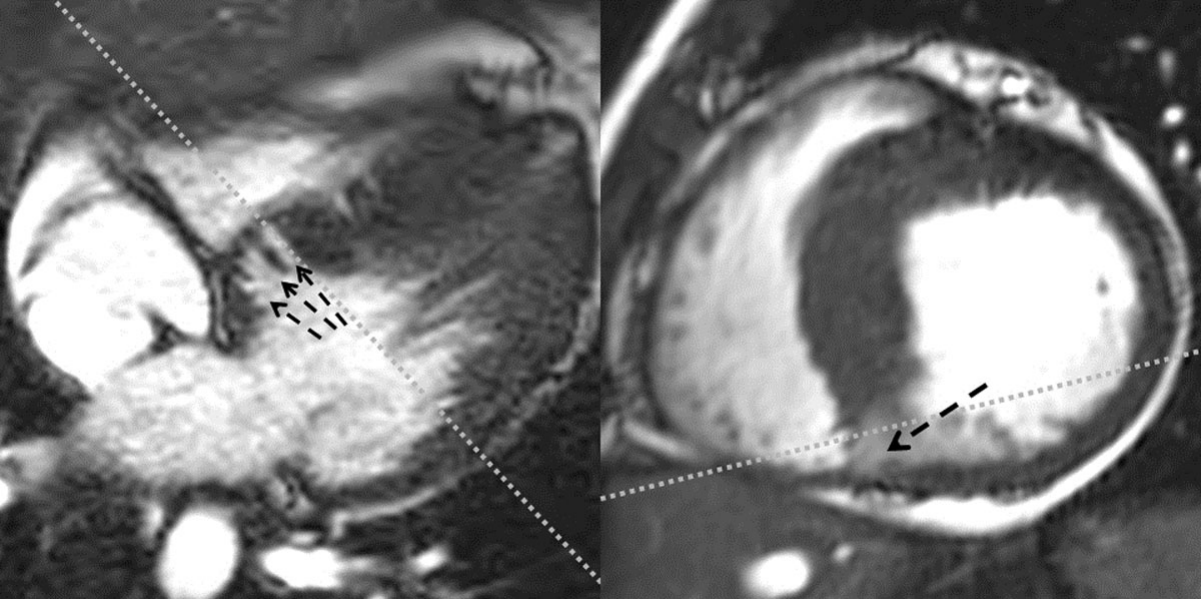
Late Diastolic Cine SSFP images acquired from a 33 year old female with a pathogenic p.Glu192Lys α-tropomyosin (TPM1) mutation demonstrating reverse septal curvature morphologic phenotype and deep basal inferoseptal crypts. The image on the left is an inferior slice from a stack of cine images acquired in 4-chamber orientation and the image on the right is a basal slice from a conventional cardiac short-axis cine stack. The grey dotted lines depict approximate planning orientation of the counterpart images.

